# Using a Bayesian Hierarchical Model for Identifying Single Nucleotide Polymorphisms Associated with Childhood Acute Lymphoblastic Leukemia Risk in Case-Parent Triads

**DOI:** 10.1371/journal.pone.0084658

**Published:** 2013-12-19

**Authors:** Ying Cao, Philip J. Lupo, Michael D. Swartz, Darryl Nousome, Michael E. Scheurer

**Affiliations:** 1 Division of Biostatistics, The University of Texas School of Public Health, Houston, Texas, United States of America; 2 Section of Hematology-Oncology, Department of Pediatrics, Baylor College of Medicine, Houston, Texas, United States of America; 3 Division of Epidemiology, Human Genetics and Environmental Sciences, The University of Texas School of Public Health, Houston, Texas, United States of America; University of Texas School of Public Health, United States of America

## Abstract

Childhood acute lymphoblastic leukemia (ALL) is a condition that arises from complex etiologies. The absence of consistent environmental risk factors and the presence of modest familial associations suggest ALL is a complex trait with an underlying genetic component. The identification of genetic factors associated with disease is complicated by complex genetic covariance structures and multiple testing issues. Both issues can be resolved with appropriate Bayesian variable selection methods. The present study was undertaken to extend our hierarchical Bayesian model for case-parent triads to incorporate single nucleotide polymorphisms (SNPs) and incorporate the biological grouping of SNPs within genes. Based on previous evidence that genetic variation in the folate metabolic pathway influences ALL risk, we evaluated 128 tagging SNPs in 16 folate metabolic genes among 118 ALL case-parent triads recruited from the Texas Children’s Cancer Center (Houston, TX) between 2003 and 2010. We used stochastic search gene suggestion (SSGS) in hierarchical Bayesian models to evaluate the association between folate metabolic SNPs and ALL. Using Bayes factors among these variants in childhood ALL case-parent triads, two SNPs were identified with a Bayes factor greater than 1. There was evidence that the minor alleles of *NOS3* rs3918186 (OR = 2.16; 95% CI: 1.51-3.15) and *SLC19A1* rs1051266 (OR = 2.07; 95% CI: 1.25-3.46) were positively associated with childhood ALL. Our findings are suggestive of the role of inherited genetic variation in the folate metabolic pathway on childhood ALL risk, and they also suggest the utility of Bayesian variable selection methods in the context of case-parent triads for evaluating the role of SNPs on disease risk.

## Introduction

Childhood acute lymphoblastic leukemia (ALL) is considered to be a condition that arises from complex etiologies involving multiple factors. The absence of consistent environmental risk factors and the presence of modest familial associations suggest ALL is a complex trait with an underlying genetic component [1]. Although previous genome-wide association studies (GWAS) and candidate gene approaches have identified susceptibility loci contributing to the genetic basis of ALL, they only explain a small fraction of the heritability [1-5]. The identification of genetic factors associated with disease is complicated by complex genetic covariance structures and multiple testing issues. Both issues can be resolved with appropriate Bayesian variable selection methods.

Bayesian variable selection methods have demonstrated remarkable performance in a variety of settings, including those with weakly collinear covariates [6,7]. Additionally, stochastic search gene suggestion (SSGS) methods combine hierarchical Bayesian models with stochastic search variable selection technology to explore the posterior distribution on the model space to make inferences about the importance of genetic loci [6,8].

SSGS has many qualities that make it a strong candidate for the identification of loci involved in genetic susceptibility. Calibrated priors provide a strong balance of power and false discovery control [6,9,10]. The hierarchical nature of the priors for variable selection allows us to easily model the biological structure of single nucleotide polymorphisms (SNPs) grouped within genes. Also, many of the studies developing and applying stochastic search variable selection have demonstrated adequate performance when modeling correlated data, such as SNPs in linkage disequilibrium (LD) [6,11]. The hierarchical nature of the model provides a means to incorporate a priori known covariance structure into the model, which can improve variable selection among multiple predictors [7]. SSGS and other Bayesian variable selection methods model disease risk in a “holistic” manner, jointly considering all SNPs in question, while balancing power and false discovery control, which is important when evaluating high-dimensional data [6,9,10].

The present study was undertaken to extend our hierarchical Bayesian model for case-parent triads to incorporate single nucleotide polymorphisms (SNPs) and incorporate the biological grouping of SNPs within genes. This approach uses conditional logistic regression likelihood to model the probability of transmission to an affected child [6]. Additionally, the case-parent triad design provides an advantage to the traditional case-control design as it is immune to population stratification bias. This is because analyses are based on whether the inheritance of alleles by affected children deviates from Mendelian expectation rather than a comparison of genotypes between a case group and a control group [12,13]. As the folate metabolic pathway is suspected to play an important role in the development of childhood ALL due to its role in the synthesis, repair, and methylation of DNA [14], we selected 128 tagging SNPs in 16 folate metabolic genes (Table 1), which is an extension of our previous assessment of folate metabolic genes and childhood ALL [15].

**Table 1:** Summary of 128 Tagging Single Nucleotide Polymorphisms.

Gene	Chromosome	Number of Tagging SNPs
*MTHFR*	1	11
*MTR*	1	16
*MTHFD2*	2	4
*MTRR*	5	13
*BHMT2*	5	4
*BHMT*	5	7
*DHFR*	5	2
*NOS3*	7	6
*FOLH1*	11	5
*FOLR2*	11	2
*MTHFD1*	14	16
*SHMT1*	17	8
*TYMS*	18	8
*CBS*	21	14
*SLC19A1*	21	4
*TCN2*	22	8

## Materials and Methods

### Study Population

The study population included 118 ALL case-parent triads recruited from the Childhood Cancer Epidemiology and Prevention Center at Texas Children’s Hospital (Houston, TX) between 2003 and 2010. Both males and females, and individuals of all racial/ethnic groups were eligible to participate. After written informed consent was obtained from the parent, we obtained a blood sample from each participant. Additionally, saliva samples were collected from parents. Participation of both parents was not required for our analysis [12]. These samples were used to obtain DNA for genotyping. Demographic and clinical data were abstracted from medical records. The study protocol was approved by the Baylor College of Medicine Institutional Review Board.

### SNP Selection and Genotyping Methods

Sixteen genes in the folate metabolic pathway (Table 1) were selected because of their role in DNA synthesis, repair, and methylation. Previous literature was also used in our selection strategy [14,16]. Tagging SNPs for the 16 genes were selected using an *r* ^2^ threshold of 0.80 and the MultiPop-TagSelect Algorithm (due to the multi-ethnic composition of the study population) in the Genome Variation Server, which utilizes information from multiple HapMap populations [17,18]. [17]SNPs with minor allele frequencies of <10% were not included in the analysis due to the sample size. Based on these criteria, 128 SNPs were available for analysis.

DNA was extracted from peripheral blood lymphocytes and saliva using the QIAmp DNA Blood Mini Kit (Qiagen, Valencia, CA) according to the manufacturer’s protocol. Genotyping was done using the Sequenom MassARRAY iPLEX platform (Sequenom, San Diego, CA) in the Human Genetics Center at The University of Texas School of Public Health according to the manufacturer’s instructions.

### Statistical Analysis

SSGS was used to analyze the ALL case-parent triad data. SSGS was specifically designed to model genetic case-parent triad data because it combines the conditional logistic regression to model the likelihood of allele transmission from parents to a diseased child with a Bayesian hierarchical model to incorporate genetic structure in variable selection [6].

#### Data Preparation and Post Processing

To prepare data for analysis using SSGS, we used SimWalk2 [19] to obtain the most likely haplotypes from linkage format data. Maximum likelihood estimators and the covariance matrix of conditional logistic regression coefficients were computed using the *clogit* command of Stata to provide necessary components for Metroppolis-Hastings sampling within SSGS. We used a modified C/C++ program SSGS [6] to sample from posterior distributions; where we modified the software to incorporate SNP data. The Markov chain output from SSGS was analyzed using the R package Bayesian output analysis (boa) [20] to obtain posterior inference and assess the convergence and stationarity of Markov chains.

#### Prior Distributions

We used a hierarchical prior distribution with two levels to model variable selection: one level for selecting genes and a second level for selecting SNPs within a gene. We assigned both the prior probability of including a gene as 0.5. These values for prior probability of inclusion have been shown to control well for both false positives and false negatives [6,9,10]. These prior settings also have the interpretation that every gene has a 50% chance of being associated with the disease and every SNP within a selected gene has a 50% chance of being associated with the disease. The prior distributions of regression coefficients were independent normal distributions with mean 0. The prior settings were chosen to best control for both false positives and false negatives [9].

#### SSGS Analysis

The SSGS analysis proceeded in two steps. In step 1, we performed SNP screening using SSGS within each chromosome. The 16 genes in the folate metabolism pathway reside on 10 different chromosomes (Table 1). Since LD typically does not span across chromosomes, we first performed independent SSGS analyses for the SNPs on each chromosome. Any SNPs with posterior probability of inclusion greater than 0.2, corresponding to Bayes factor greater than 0.75, proceeded to step 2. We used this threshold for posterior probability of inclusion, recognizing that we wanted to include as many SNPs in the second step as possible, even if the posterior evidence is mildly in favor of non-inclusion. In step 2, all the SNPs selected from step 1 were analyzed simultaneously to identify SNPs associated with childhood ALL. Posterior inference, including odds ratios (ORs) and their 95 percent credible intervals (95% CIs), were calculated for SNPs with Bayes factor greater than 1. For each SSGS analysis, we ran two chains with different initial values for 600,000 iterations and used the last one-third iterations of two chains for pooled posterior inference. All the Markov chains passed the Geweke convergence diagnostic and the Heidelberger-Welch test for stationary using R package boa. In addition, two chains from different initial values for each analysis had high correlations, indicating convergence to the same posterior distribution.

## Results

The population characteristics of childhood ALL cases included in our study were summarized in Table 2. There were 118 cases recruited from Texas Children’s Cancer Center from 2003 to 2010, including 65 males and 53 females. For the study period, the participation rate was 85%. Of the 118 families, 36% were complete triads, however, based on previous assessments this is unlikely to bias the results [12]. The study included individuals of all racial/ethnic groups, 59 non-Hispanic whites, 6 non-Hispanic blacks, 46 Hispanics, and 7 belonging to other racial/ethnic groups. All the cases were under 14 years old when recruited.

**Table 2:** Population characteristics of childhood acute lymphoblastic leukemia cases, Texas Children’s Cancer Center, 2003-2010.

Characteristic	No. (%)
Triads included	118
Case sex	
Male	65 (55.0)
Female	53 (45.0)
Race/ethnicity	
Non-Hispanic White	59 (50.0)
Non-Hispanic Black	6 (5.1)
Hispanic	46 (39.0)
Other	7 (5.9)
Age (range 0-14 years)	
<4 years	56 (47.4)
4-7 years	46 (39.0)
>7 years	16 (13.6)

We analyzed 128 tagging SNPs of 16 folate metabolic genes to identify SNPs associated with childhood ALL. The SNPs with posterior probability of inclusion greater than 0.2 in the initial screening within each chromosome proceeded to the final analysis. In the initial screening, 7 SNPs among the 128 tagging SNPs were identified, 2 SNPs in gene *MTHFD2*, 3 SNPs in gene *BHMT2*, 1 SNP in gene *NOS3*, and 1 SNP in gene *SLC19A1* (Table 3).

**Table 3:** SNPs Selected from Initial Screening.

Gene	SNP	PPI* from chromosomal analysis (Phase 1)	Bayes factor from joint analysis (Phase 2)
*MTHFD2*	rs1667627	0.335	0.341
*MTHFD2*	rs12196	0.227	0.268
*BHMT2*	rs670220	0.245	0.576
*BHMT2*	rs682985	0.449	0.580
*BHMT2*	rs557302	0.405	0.311
*NOS3*	rs3918186	0.854	7.381
*SLC19A1*	rs1051266	0.216	1.926

^*^ Posterior probability of inclusion.

In the final analysis of 7 selected SNPs from initial screening, *NOS3* rs3918186 and *SLC19A1* rs1051266 had Bayes factors greater than 1 (Table 4). In other words, the posterior odds of including the two SNPs in the model were greater than the prior odds of including the two SNPs, indicating that our data supported the association between the two SNPs and childhood ALL risk. Specifically, *NOS3* rs3918186 had a Bayes Factor of 7.38, whereby for each copy of the minor allele, there was a 2.16 times risk of developing childhood ALL (OR = 2.16; 95% CI: 1.51-3.15) [21]. We found less evidence in our data for *SLC19A1* rs1051266 being associated with childhood ALL. For each copy of the minor allele of *SLC19A1* rs1051266, there was a 2.07 times risk of developing childhood ALL (OR = 2.07; 95% CI: 1.25-3.46).

**Table 4:** Two SNPs Identified from Final Analysis.

Gene	SNP	Odds ratio	95% CI	PPI*	Bayes factor
*NOS3*	rs3918186	2.16	(1.51, 3.15)	0.711	7.38
*SLC19A1*	rs1051266	2.07	(1.25, 3.46)	0.391	1.93

^*^ Posterior probability of inclusion.

## Discussion

To our knowledge, this is the first application of Bayesian hierarchical models designed for case-parent triads to identify SNPs associated with disease. Previous assessments utilizing this approach have explored microsatellite markers [6]. We extended this method to include the evaluation of SNPs because of the availability of high-dimensional SNP array data for many phenotypes and as case-parent triads are becoming more common in the role of inherited genetic variation on childhood cancer risk [15,22-25]. The case-parent triad design provides an advantage to the traditional case-control design as it is immune to population stratification bias. This is because analyses are based on whether the inheritance of alleles by affected children deviates from Mendelian expectation rather than a comparison of genotypes between a case group and a control group [12,13]. Additionally, the case-parent triad design is useful when appropriate controls are difficult to identify or enroll. Finally, family-based designs often provide greater power than traditional case-control designs [26].

In our study, we used SSGS to analyze 128 tagging SNPs in 16 folate metabolic genes to identify associations with childhood ALL risk. Using Bayes factors among this variants in childhood ALL case-parent triads, two SNPs were identified with a Bayes factor greater than 1. There was evidence that the minor alleles of *NOS3* rs3918186 and *SLC19A1* rs1051266 were positively associated with childhood ALL according to commonly cited guidelines for Bayes Factors [21]. In fact, the minor alleles of each of these SNPs carried twofold increase in risk for childhood ALL.

Endothelial nitric oxide synthase 3 (NOS3) is responsible for the production of nitric oxide (NO), which modulates homocysteine concentrations by inhibition of 5-methyltetrahydrofolate-homocysteine methyltransferase, the enzyme that synthesizes methionine from homocysteine and 5-mTHF [27]. *NOS3* rs3918186 is an intronic variant [28]. Nitric oxide and oxidative stress have been suggested as potential mechanisms of childhood leukemogenesis [14,16,23,29,30]. To our knowledge, this variant has not been assessed in relation to childhood ALL.

The gene *SLC19A1* encodes for reduced folate carrier 1 (RFC-1), which is a transmembrane protein that transports folate across cell membranes, thereby influencing folate levels. The G80A (rs1051266, G>A) polymorphism in RFC-1 is associated with altered folate/antifolate levels [31,32]. While, to our knowledge, *SLC19A1* rs1051266 has not been evaluated for childhood ALL risk, it has been associated with colorectal cancer [33] and prostate cancer [34]. However, a recent assessment by Metayer et al. evaluating *SLC19A1*, as well as other genes in the folate pathway, used a tagging SNP approach found no association with SNPs in *SLC19A1* and childhood ALL [16].

The major limitation of this study is the sample size (*n* = 118), which did not allow us to detect modest associations. In fact, based on this sample size, with a minor allele frequency of 10% (our minor allele frequency inclusion criteria for SNPs), α=0.05, β=0.8, and assuming a log-additive model of inheritance, we had the power to detect an odds ratio of 2.12 based on power calculations using Quanto Version 1.2.5 [35-37]. Our SNP selection strategy may have also affected our ability to identify associations, as we limited our inclusion to those with a minor allele frequency of ≥10%. In other words, we were not able to discover disease associations due to rare variants. Additionally, we were not able to stratify our results by ALL subtypes (e.g., B-lineage or T-lineage), as this information was not available, or age at diagnosis. However, in spite of these limitations, we were able to identify significant associations between folate metabolic variants and childhood ALL using SSGS. An important strength of our study was the use of the case-parent triad design. Additionally the use of SSGS allowed for the control for multiple comparisons [9], which is important as we evaluated 128 SNPs.

Our findings are suggestive of the role of inherited genetic variation in the folate metabolic pathway on childhood ALL risk. We believe they also suggest the utility of Bayesian variable selection methods in the context of case-parent triads for evaluating the role of SNPs on disease risk, especially under the circumstances of smaller sample sizes. We identified two potential inherited effects that were undetected in our previous study [15]. Our findings suggest that SSGS can be used to incorporate LD information to identify disease associated SNPs and to appropriately estimate the relative risk coefficients through averaging the posterior distributions [6,10]. Additionally, as we evaluated 128 SNPs, the use of the priors used here have been shown to control for false positive findings in simulation studies [9]. The use of Bayes factors offers a way to summarize the strength of evidence in our data for specific SNPs [21], allowing us to prioritize future follow-up investigations. Overall, SSGS provides a useful approach to investigate genetic factors associated with early onset diseases such as childhood ALL.
